# Assessment of Physical Work Demands of Home Care Workers in Norway: An Observational Study Using Wearable Sensor Technology

**DOI:** 10.1093/annweh/wxac052

**Published:** 2022-08-12

**Authors:** Svein O Tjøsvoll, Øystein Wiggen, Victor Gonzalez, Trine M Seeberg, Skender Elez Redzovic, Ingeborg Frostad Liaset, Andreas Holtermann, Marius Steiro Fimland

**Affiliations:** Department of Neuromedicine and Movement Science, Faculty of Medicine and Health Sciences, NTNU Norwegian University of Science and Technology, Trondheim, Norway; Health Research, SINTEF DIGITAL, SINTEF AS, Trondheim, Norway; Smart Sensor Systems, SINTEF DIGITAL, SINTEF AS, Oslo, Norway; Smart Sensor Systems, SINTEF DIGITAL, SINTEF AS, Oslo, Norway; Department of Neuromedicine and Movement Science, Faculty of Medicine and Health Sciences, NTNU Norwegian University of Science and Technology, Trondheim, Norway; Department of Neuromedicine and Movement Science, Faculty of Medicine and Health Sciences, NTNU Norwegian University of Science and Technology, Trondheim, Norway; National Research Centre for the Working Environment, Lerso Parkalle, Copenhagen, Denmark; Department of Neuromedicine and Movement Science, Faculty of Medicine and Health Sciences, NTNU Norwegian University of Science and Technology, Trondheim, Norway; Unicare Helsefort Rehabilitation Centre, Rissa, Norway

**Keywords:** manual labor, physical exposures, occupational physical activity, human factors, ergonomics, occupational health and safety, accelerometry, heart rate monitor

## Abstract

**Objectives:**

High physical work demands are believed to be partly responsible for the high sickness absence among home care workers, but no studies have assessed their physical work demands using precise device-based measurements. Hence, the objective of this observational study was to assess physical work demands in home care, using wearable sensors.

**Methods:**

From six home care units in a large municipality in Norway, 114 of 195 eligible home care workers filled in a questionnaire, a diary about work hours, and wore five accelerometers, and a heart rate sensor for up to six consecutive workdays.

**Results:**

On average, the homecare workers spent 50% of the working hours sitting, 25.2% standing, 11.4% moving, 8.3% walking fast, 1.9% walking slow, 1.2% stair-climbing, 0.3% cycling, and 0.05% running. We found the following exposures to demanding postures: arm-elevation in an upright body position ≥30° was 36.7%, ≥60° was 4.1%, and ≥90°was 0.5%; forward trunk inclination in an upright body position ≥30° was 9.9%, ≥60° was 4%, and ≥90° was 1%; and for kneeling it was 0.8%. We found the average cardiovascular load (%heart rate reserve) during work to be 28%. There was considerable individual variation in these physical exposures at work.

**Conclusions:**

This study presents precise information on various physical work demands of home care workers in Norway. Home care workers spent on average half the workday sitting and the remaining time in various occupational physical activities. Presently, few device-based exposure limits have been proposed for acceptable amounts of occupational physical exposures, but the level of arm-elevation, forward trunk inclination, and the considerable variation of physical workloads among home care workers, indicate that preventive measures should be taken.

**What’s Important About This Paper?**

This observational study presents detailed information on objectively measured physical work demands of home care workers in Norway. The findings extend our understanding of occupational ergonomic risk factors and can contribute to improved occupational health and safety measures and interventions in home care.

## Introduction

Home care workers assist individuals that need help with activities of daily living due to a disability, impaired health, or disease. This requires the home care worker to perform various work tasks in caregiving to patients that can be physically demanding, such as prolonged standing and walking, lifting, carrying, pushing, and pulling. These work tasks can also often involve challenging work postures, such as bending forward, elevated arms, and kneeling (Chappel *et al.*, 2017; van der Molen *et al.*, 2017; Coenen *et al.*, 2018b; Lunde *et al.*, 2019; Wang *et al.*, 2020). While there is ample evidence that leisure-time physical activity is good for health, occupational physical activity has been associated with several adverse health outcomes (Coenen *et al.*, 2018a; Holtermann *et al.*, 2021). These prolonged work exposures are known risk factors for musculoskeletal disorders, injuries, sick leave, and early retirement (Holtermann *et al.*, 2021; Hulshof *et al.*, 2021b). Accordingly, home care workers (i.e. nurses, nursing assistants, learning disability nurses, and occupational therapists) perceive their work as exhausting and physically demanding (Delp *et al.*, 2010). Alongside organizational factors, autonomy at work, and psychosocial variables (Aronsson *et al.*, 2017), the mentioned physical work demands could contribute to health complaints and adverse effects in home care workers (Carneiro *et al.*, 2017; Hulshof *et al.*, 2021a), as it does in many workers with high occupational physical activity (Fimland *et al.*, 2018). This could explain some of the high sickness absence in home care of around 11% in Norway, which is higher than for the health care sector in general (9.7%), and considerably higher than across all occupations (6.0%) (StatisticsNorway, 2021).

Due to the increasing number of older adults in need of care and the lack of qualified personnel, it is of increasing importance that home care workers have working conditions that are not detrimental to health. To identify how it is feasible to improve the physical working conditions, it is necessary to first generate precise knowledge of the current physical work demands in this vulnerable occupational group.

Self-reported methods, and visual- and video-based observations have been the primary tools for assessing physical work demands, but these methods are either highly inaccurate, less cost-effective, or time consuming (Koch *et al.*, 2016; Gupta *et al.*, 2017). Device-based measurements of occupational physical activity and postures using inertial measurement units have become more extensively utilized. Development in microelectromechanical systems technology enables feasible long-term measurements of physical exposures, with a high level of accuracy (Duncan *et al.*, 2018; Stewart *et al.*, 2018; Twomey *et al.*, 2018).

However, assessment of physical work demands using device-based measurements during work is limited (Koch *et al.*, 2017; Loef *et al.*, 2018; Jorgensen *et al.*, 2019; Lunde *et al.*, 2019), and few studies have applied technical measurements in quantifying physical work demands in employees in healthcare (Loef *et al.*, 2018; Merkus *et al.*, 2019). Furthermore, no studies have used wearable sensors to measure physical work demands in home care workers. Thus, the purpose of our study was to assess physical exposures in home care workers in a large Norwegian municipality using wearable sensors. Specifically, we used accelerometers and heart rate sensors to assess physical exposures, demanding postures, and cardiovascular load over several workdays.

## Materials and methods

### Study population

Home care workers with ≥50% employment (minimum 18.8 working hours a week) were recruited from six of a total of 13 home care service units in Trondheim, the third largest city in Norway. Only workers that had direct contact with patients were included. All workers in these home care units were provided written and oral information about the research project and gave written consent before the study. Exclusion criteria were: (1) physical disability not allowing normal behavior, (2) office work, (3) bandage band aid and adhesives allergy, and (4) pregnancy. The study was conducted according to the Declaration of Helsinki and approved by the Regional Committees for Medical Research Ethics—Central Norway (No.: 64541).

### Anthropometrics

Baseline measurements of height and weight were collected using a standardized digital body weight scale and a wall-mounted SECA 206 measuring tape (SECA Medical Measuring Systems and Scales, Birmingham, UK).

### Sensor measurements

Five triaxial AX3 accelerometers (Axivity Ltd, Newcastle upon Tyne, UK) were mounted on the skin of the home care workers, using adhesive double-sided tape (3M; Witre, Halden, Norway) and secured with waterproof medical tape (Opsite Flexifix; Mediq, Oslo, Norway). They were worn 24 h per day for up to six consecutive workdays at a sampling frequency of 25 Hz and a range of ±8 G. The accelerometers were attached to the following anatomical locations: (1) below the head of the fibula, on the proximal and lateral aspect of the calf, (2) on the distal, anterior and medial aspect of the femur (approximately 10 cm above the crest of the patella), (3) below the iliac crest of the hip, (4) the upper back approximately 5 cm to the side of the processus spinosus at the level of Th1–Th2 vertebrae, and (5) on the upper arm, approximately at the insertion of the deltoid muscle (Korshoj *et al.*, 2014; Skotte *et al.*, 2014; Hallman *et al.*, 2015). Sensors were mounted on the calf for classification of kneeling, on the thigh and hip for classification of sitting, standing, moving, walking, running, stair-climbing, and cycling, on the upper back for classification of forward trunk inclination, and on the upper arm for classification of arm-elevation. The sensors on the extremities were mounted on the dominant side of the participants. Participants received a paper activity diary to fill in daily: (1) when they got up in the morning, (2) sensor calibration jump, (3) arrived at work, (4) finished at work, and (5) when they went to sleep.

Heart rate was assessed using the Firstbeat Bodyguard 2 monitor (Firstbeat Technologies Ltd., Jyväskylä, Finland) (Parak *et al.*, 2015) and was measured 24 h up to 6 days, detecting the beat-to-beat intervals with a sampling frequency of 1000 Hz. Single-use and pre-gelled electrocardiography electrodes (Arbo H92SG) were mounted on the chest of the participants. This sensor had to be removed prior to activities in water and attached afterward by participants.

### Data collection

Data from questionnaires, anthropometric measurements, cardiorespiratory fitness test, and technical measurements were collected from September 2020 to April 2021. All data were stored and analyzed according to current guidelines for data protection (GDPR.EU, 2021). Prior to participation, each participant filled out a questionnaire regarding sociodemographic, health-related, and workplace factors (NTNU, 2021).

### Aerobic workload

The aerobic workload was measured as percent heart rate reserve (HRR) and was calculated using the following equation(Karvonen *et al.*, 1957):


$$\begin{array}{l} \%\;HRR=\frac{HR_{HRwork}-HR_{HRmin}\;}{HR_{HRmax\;}-HR_{HRmin}}\times 100\;\% \end{array}$$

Calculation of HR_min_ was conducted using a moving window over an average of 10 beats for the lowest total heart beats every night, across the measurement period of every worker. HR_max_ was estimated according to (Tanaka *et al.*, 2001):


$$\begin{array}{l} HR_{max}=208-0.7\;\times Age \end{array}$$

The maximal %HRR was calculated from the average of the highest measured heart rate across all workdays, whereas the mean %HRR was calculated from all values across workdays.

### Assessment of cardiorespiratory fitness

Maximal aerobic capacity $\left(\dot{V}O_{2max}\right)$ was estimated using the submaximal Ekblom-Bak test (Björkman *et al.*, 2016; GIH, 2021). The test was conducted on the following cycle ergometer models: Monark 839E or Monark 939E (Monark AB, Varberg, Sweden). Heart rate during this test was measured using Polar H10 or Garmin HRM-dual heart rate sensor chest strap.

### Data processing

Data from the questionnaire was manually transferred from paper to a spreadsheet for further processing. AX3 sensors were configured using the software OMGUI (version 1.0.0.43; Axivity Ltd, Newcastle upon Tyne, UK). The data was manually downloaded and processed using a modified version of the custom-made MATLAB software (Acti4, The National Research Centre for the Working Environment, Copenhagen Denmark) (Stemland *et al.*, 2015). Using rule-based models, the software was able to determine activity categories and postures such as lying, sitting, standing, moving, walking slow, walking fast, running, cycling, stair-climbing, arm-elevation, forward trunk inclination, and kneeling with a high level of sensitivity and specificity (Korshoj *et al.*, 2014; Skotte *et al.*, 2014; Hallman *et al.*, 2015; Hendriksen *et al.*, 2020; Tjøsvoll *et al.*, 2022). The Acti4 software classified non-wear time if there was no movement during periods longer than one and a half hours in non-sleep periods. Activity diaries were manually plotted in the Acti4 software for each participant into the following categories: (1) sensor calibration, (2) working hours, (3) after working hours, and (4) sleep. A batch analysis was performed, and all the data were imported to a csv file.

Python (version 3.9.5; Python Software Foundation 2001-2021) was used to derive working hours from the dataset. A minimum of two working days was considered valid, and a cutoff of ≥4 h was set for all workdays for each worker to be eligible for inclusion in further analysis (Jorgensen *et al.*, 2019). Heart rate was downloaded using the Firstbeat Uploader software (Firstbeat Technologies Ltd., Jyväskylä, Finland) with default settings and processed together with the accelerometer data using the Acti4 software. Heart rate-data containing errors ≥50% were removed from the dataset. Two participants were removed from arm-elevation and forward trunk inclination due to technical error of the sensor on the upper- arm and back. One participant was removed from mean HRR, due to missing values. The average HRR for each participant was calculated by adding HRR values from all valid workdays for each participant.

### Statistics

The following descriptive statistics were calculated for all workers: weighted mean, standard deviations (SD), and percentages. The statistical processing of the data was conducted in Python using the code editor Spyder for data analysis. Furthermore, the statistical libraries Pandas version 1.2.4, Seaborn version 0.11.1. NumPy, Matplotlib.pyplot, and the OS module were imported, allowing scientific computing.

## Results

**Figure 1.**
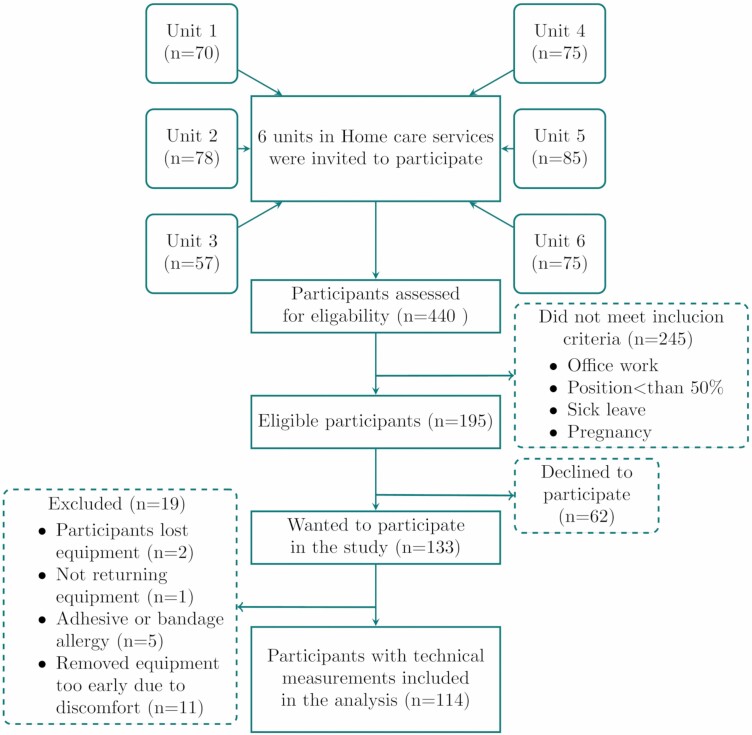
Flow of participants.

For an average of 3.8 workdays, 2913 h of accelerometer data and 2826 h of heart rate data were recorded in 114 home care workers. The flow of participants can be seen in Fig. 1. In brief, 195 of 440 home care workers were eligible to participate in this study. Demographics, health, and work characteristics of the 114 home care workers that completed the study are depicted in Table 1.

**Table 1.** Demographics, health, and work characteristics of home care workers (*N* = 114).

Demographic characteristics	%	Mean (SD) ^a^	*N*
Age (years)	100	36.7 (12.4)	114
Gender	100		114
Female	71		81
Male	28.9		33
Cardiorespiratory fitness^b^ (ml/kg/min)	100		114
Female		34.4(8.5)	81
Male		44(9.5)	33
Body mass index (kg/m^2^)	100		114
Female		26.9 (5.2)	81
Male		26.3 (3.7)	33
Marital status	98.3		112
Married/partner	24.6		28
Not married/living alone	73.7		84
Education (years)	100		114
High school (up to 3 years)	13.1		15
Certificate of completed Apprenticeship	24.6		28
College/university	62.3		71
Worked in home care(years)	98.2	6.4 (7.3)	112
Work demands^c^	100		114
Work requires you to work fast		3.5(0.6)	114
Work requires you to work hard		3 (0.6)	112
Work requires too much effort		3.1 (0.7)	113
Work requires ingenuity		3 (0.6)	112
Decide how to perform your work tasks		3.2(0.7)	114
Decide your own work tasks		2.6(0.8)	113
Tired after work		2.7 (0.8)	113
Work ability index^d^	95.6	8.6(1.4)	109
Perceived health^e^	99.1	2.2(0.6)	113
Pain at least 3 months during the last year	93.9		107
Experienced pain at least 3 months during the last year	58.8		67
Did not experience pain at least 3 months during the last year	35.1		40
Pain regions for at least 3 months during the last year	36		41
Jaw	9.6		11
Neck	36		41
Shoulders	35.1		40
Elbow	5.3		6
Wrist and fingers	10.5		12
Chest	6.1		7
Upper-back	19.3		22
Lower back	34.2		39
Hip	9.6		11
Thighs	2.6		3
Knees	15.8		18
Calves	7		8
Feet and ankles	10.5		12
Prevented activities during work because of this pain	18.4		21
Sick leave last 12 months	71.9		82
<2 weeks	42.1		48
>2 weeks	29.8		34
Smoking habits	90.4		103
Have smoked or currently smoking	40.4		46
Never smoked	50		57
Self-reported leisure time physical activity	88.6		101
Never	1.8		2
Once a week	10.5		12
2–3 times a week	49.1		56
Every day	27.2		31

^a^SD = standard deviation;

^b^ $\dot{V}O_{2max}$ ;

^c^ 1 = never, 2 = no/rarely, 3 = yes/sometimes 4 = yes/frequently;

^d^ work ability index, 0 = cannot work, 10 = best;

^e^ 1 = poor, 2 = not that good, 3 = good, 4 = very good.

The home care workers consisted of nurses, nursing assistants, learning disability nurses, and occupational therapists, having home care as their main employer, and worked an average of 38.5 h a week. According to the self-reported measurements, 46 (40.4%) of the home care workers reported that they persistently felt fatigued.

**Figure 2.**
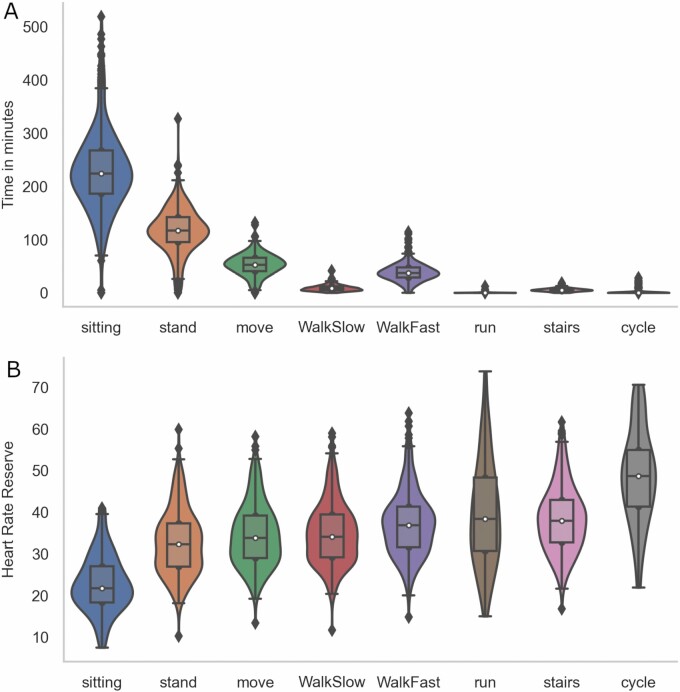
Time in minutes spent in occupational physical activity (A) and the corresponding %HRR (B). The violin plots depict information about the distribution of the data. The box displays the median, 25th and 75th percentile and the black lines are showing the rest of the distribution.

Meantime in percent and minutes spent in physical exposures and demanding postures at work, standard deviations, mean %HRR and the relation between HRR and physical exposures at work are depicted in Fig. 2 and Table 2, respectively. Most of the workday was spent sitting (50%) and occupational physical activity (48%), and included standing (25.2%), moving (11.4%), walking slow (1.9%), walking fast (8.3%), running (0.05%), stair climbing (1.2%), and cycling (0.3%). The average %HRR was highest for cycling (48.5%), running (39.3%), stair climbing (38.4%), and walking fast (37.2%). The lowest %HRR was measured for sitting (22.9%). The average %HRR for the remaining OPA-categories were standing (33.7%), moving (34.5%), and walking slow (34.9%).

**Table 2.** Physical exposures of home care workers during working hours (*N* = 114).

Occupational physical activity	Time in %	SD	Time (min)	SD	Mean %HRR	SD
Sitting	49.6	15.2	232.9	78.6	22.9	6.8
Standing	25.2	8.9	117.6	45.1	32.7	7.7
Moving	11.4	4.2	53.2	21.2	34.5	7.5
Walking slowly	1.9	1.2	8.8	5.3	34.9	7.4
Walking fast	8.3	3.5	39	18.1	37.2	7.7
Running	0.0	0.2	0.2	0.8	39.3	13.4
Stair-climbing	1.2	0.7	5.5	3.2	38.4	8
Cycling	0.3	1	1.4	4.4	48.5	11.4
Light intensity physical activity	38.4	12.6	179.7	64.5	33.4	7.6
Moderate to vigorous physical activity	9.9	4.1	46.2	20.4	40.6	8.3
Total activity	48.3	15.3	225.8	79.3	36.1	8.7

Demanding postures	Time in %	SD	Time (min)	SD	—	—
Kneeling	0.8	1.3	3.9	5.8	—	—
Arm-elevation ≥30°	36.8	13.4	171.4	64.2	—	—
Arm-elevation in an upright body position ≥30°	30.7	12.4	143.7	59.8	—	—
Arm-elevation ≥60°	4.9	3.9	22.4	16.1	—	—
Arm-elevation in an upright body position ≥60°	4.1	3.3	18.8	14.4	—	—
Arm-elevation ≥90°	0.7	0.8	3.2	3.2	—	—
Arm-elevation in an upright body position ≥90°	0.5	0.7	2.5	2.7	—	—
Forward trunk inclination ≥30°	22.2	12	103.1	56.5	—	—
Forward trunk inclination in an upright body position ≥30°	9.9	5.8	46.2	27.9	—	—
Forward trunk inclination ≥60°	5	5.2	22.7	18.8	—	—
Forward trunk inclination in an upright body position ≥60°	3.9	2.6	18.3	12.4	—	—
Forward trunk inclination ≥90°	1.3	4.4	5.6	13.3	—	—
Forward trunk inclination in an upright body position ≥90°	1	1.5	4.7	5.9	—	—

Values are means and standard deviations (SD). *N*: number, %HRR: %heart rate reserve, LIPA: light intensity physical activity (standing, moving, and walking slow), MVPA: moderate to vigorous physical activity (walking fast, running, stair-climbing, and cycling), total activity (standing, moving, walking slow, walking fast, running, stair-climbing, and cycling).

**Figure 3.**
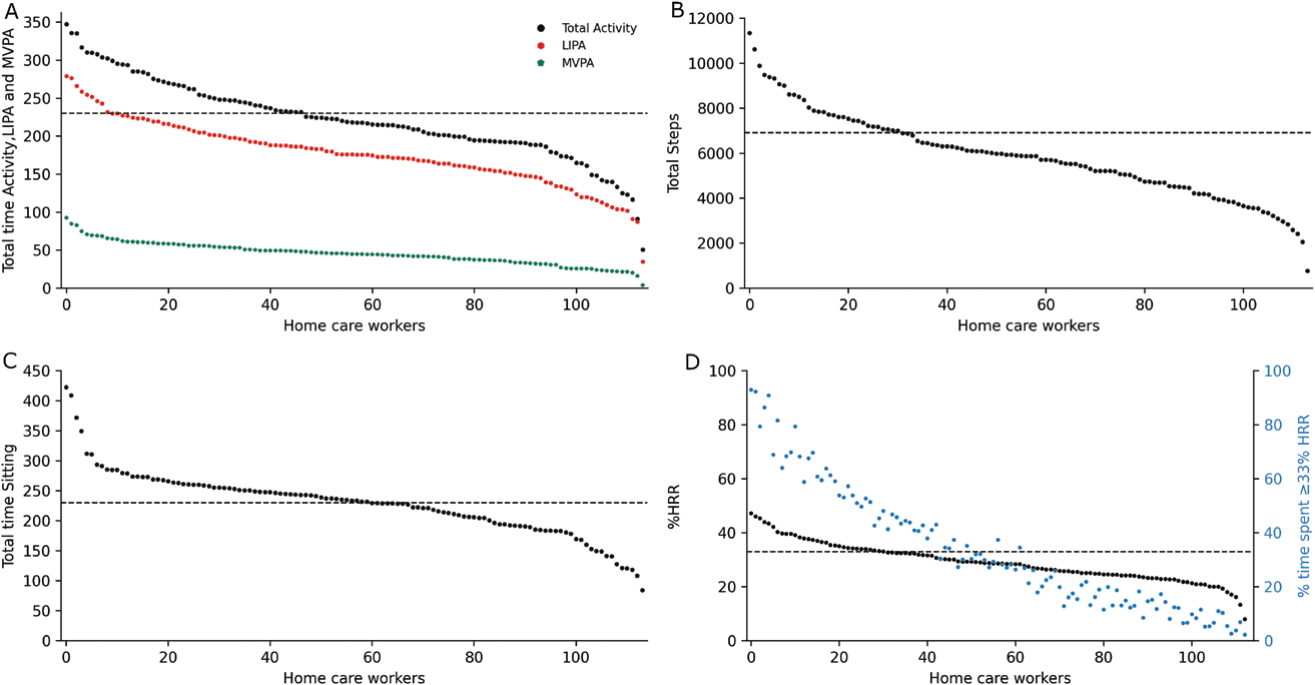
Occupational physical activity on an individual level. Home care workers are represented during working hours on the *x*-axis and on the *y*-axis (A) Minutes in LIPA (light intensity physical activity: standing, moving, and walking slow) and MVPA (moderate to vigorous physical activity: walking fast, running, stairclimbing, and cycling) and total activity (LIPA+MVPA); (B) total steps for each home care worker; (C) total minutes sitting during working hours; and (D) mean %HRR for each home care worker and time in percentage spent with at least 33% HRR. The black horizontal dotted lines in A–C depicts the threshold for 50% of workday and in D the threshold for 33% HRR.

**Figure 4.**
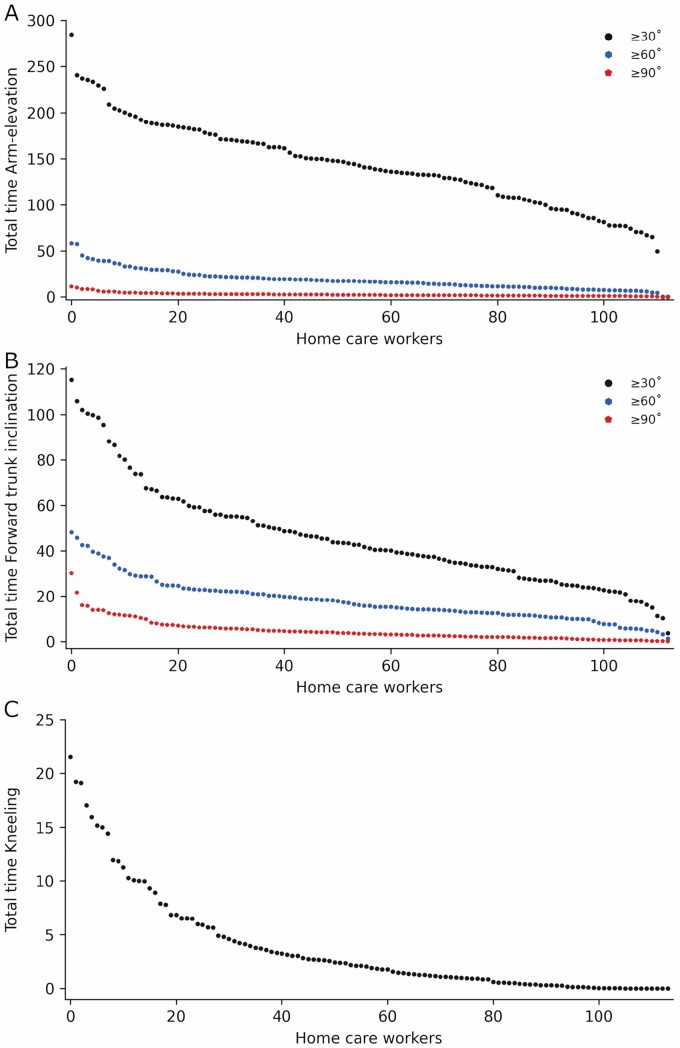
Demanding postures on an individual level. Home care workers are represented during working hours on the *x*-axis and on the *y*-axis (A) total minutes arm-elevation ≥30°, ≥60°, and ≥90° in an upright body position, (B) total minutes ≥30°, ≥60°, and ≥90° forward trunk inclination in an upright body position and (C) total minutes kneeling.

Fig. 3 depicts physical work demands, while Fig. 4 depicts the time in various demanding postures, on an individual level during the workday.

## Discussion

Home care workers had an average aerobic workday load of 28% HRR, spent half the workday sitting, and the other half in the following activities: 25% standing, 11% moving, 8% walking fast, 2% walking slow and very little in stair-climbing, cycling, and running. Regarding demanding postures: arm-elevation in an upright body position ≥30° occurred 31% of the workday, ≥60° was 4%, and ≥90° was 0.5%; forward trunk inclination in an upright body position ≥30° was 10%, ≥60° was 4% and ≥90° was 1%; and for kneeling it was 0.8%. Furthermore, our results indicate an uneven distribution of physical exposures among home care workers.

Most studies of physical work demands have used self-reported assessment methods, known for being imprecise and prone to bias (Gupta *et al.*, 2017). When comparing our findings with other objective evaluations of workplace physical exposures which used similar methods, we found that home care workers spent less time sitting and more time on their feet (50% sitting versus 47% on feet) than transportation workers in Denmark (58% versus 34%) (Jorgensen *et al.*, 2019), non-shift working nurses in Netherland (64% versus 36%) (Loef *et al.*, 2018) and white-collar workers in Denmark (66% versus 34%) (Jorgensen *et al.*, 2019), but more sitting and less time on feet than childcare workers in Denmark (45% versus 51%) (Holtermann *et al.*, 2020), workers in manufacturing in Denmark (30% versus 70%) (Jorgensen *et al.*, 2019) and cleaners in Denmark (23% versus 78%) (Jorgensen *et al.*, 2019). For home care workers, the average cardiovascular workday-strain and number of steps (28% HRR; 6896 steps) was higher than for white-collar workers (23%; 4229) (Jorgensen *et al.*, 2019) but lower than for workers in manufacturing (30%; 7885) (Jorgensen *et al.*, 2019), cleaning (34%; 10149) (Jorgensen *et al.*, 2019), and transport (32%; 8910) (Jorgensen *et al.*, 2019). The amount of moderate to vigorous physical activity (i.e. walking fast, running, stair climbing, and cycling) accounted for a small part of the workday with an average of 7 min or 1.6% of the home care workday. This was still more than for white-collar workers (1.9 min) (Jorgensen *et al.*, 2019), manufacturing workers (3 min) (Jorgensen *et al.*, 2019), non-shift working nurses (4 min) (Loef *et al.*, 2018), childcare workers (4.2 min) (Holtermann *et al.*, 2020), cleaners (5.4 min) (Jorgensen *et al.*, 2019), and similar as transportation workers (6.9 min) (Jorgensen *et al.*, 2019).

Regarding demanding postures, home care workers were exposed to a lower percentage of work time of arm-elevation while standing (≥60° of 4.1% and ≥90° of 0.5%) than childcare (≥60° of 5% and ≥90° of 1.1%) (Holtermann *et al.*, 2020), cleaning (≥60° of 5.7% and ≥90° of 1%) (Jorgensen *et al.*, 2019), manufacturing (≥60° of 6.3% and ≥90°of 1.3%) (Jorgensen *et al.*, 2019), and transportation workers (≥60° of 10% and ≥90° of 1.2%) (Jorgensen *et al.*, 2019). The exposure levels for ≥30° of 9.9% and ≥60° of 3.9% forward trunk inclination in an upright body position in home care were about the same as childcare (≥30° of 10.6%; and ≥60° 4.3%) (Holtermann *et al.*, 2020). Furthermore, home care workers were conducting almost similar amounts of ≥60° of 3.9% and ≥90° of 1% forward trunk inclination as cleaning (≥60° of 3.8% and ≥90°of 1%) (Jorgensen *et al.*, 2019), transport (≥60° of 3.6% and ≥90°of 1%) (Jorgensen *et al.*, 2019), and manufacturing workers (≥60° of 3% and ≥90°of 0.8%) (Jorgensen *et al.*, 2019). No information was provided on ≥30° arm-elevation for all sectors and information about ≥30° forward trunk inclination was only available for childcare, whereas ≥90° forward trunk inclination was not included in the childcare study. There were only comparable data of objective measurements of kneeling for childcare workers (2.5%) (Holtermann *et al.*, 2020), conducting more work kneeling than we found in home care workers (0.8%).

On an average home care workday, about half was conducted sitting and the other half in occupational physical activity, whereof one-fourth was spent standing and one-fourth doing various movements. Recent research indicates that physical exposures and their impact on health is domain specific due to different characteristics (Gupta *et al.*, 2020; Ketels *et al.*, 2020). Whereas leisure time physical activity provides beneficial health effects, a growing body of evidence has demonstrated that a high level of occupational physical activity is associated with a higher risk of sickness absence, cardiovascular disease, musculoskeletal disorders, and all-cause mortality (Coenen *et al.*, 2018a; Holtermann *et al.*, 2021)—and is referred to as the physical activity health paradox. However, it is presently not well understood what constitutes a healthy composition of physical exposures at work or which combination provides the most favorable balance between physical exposures and rest. Nevertheless, sitting is not necessarily indicative of physical rest, driving, or desk work as indicated by 23% HRR. Several core tasks are commonly performed while sitting in home care (e.g. documentation-work, putting compression stockings on patients, wound care, dressing, feeding, and activation activities). In addition, home care workers were on average within the recommended threshold proposed by the International Labor Organization (ILO) of 33% HRR (Bonjer, 1971; Jørgensen, 1985; Wu and Wang, 2002; Brighenti-Zogg *et al.*, 2016). However, we cannot eliminate the possibility that when exposed to cardiovascular workloads above the proposed threshold, repeated throughout days, weeks, months, and years, could impose a risk on the health of these workers.

The high amount of arm elevation ≥30° we observed in home care workers, has been associated with a more than two-fold risk of sickness absence in a recent study (Gupta *et al.*, 2022), and 36% of the home care workers reported long-term neck/shoulder pain in the past year. Furthermore, another known occupational risk factor is forward trunk inclination, which was prevalent in home care workers, and coincides with 34% reporting long-term low back pain. A 2-year follow-up study using device-based measurement methods conducted by the National Institute of Occupational Health in Norway found an association between ≥30° forward trunk inclination and low back pain intensity in health care workers (Lunde *et al.*, 2019). These results indicated that forward trunk inclination ≥30° in an upright body position for ≥100 min was associated with an increase in low back pain intensity of 0.8–0.9 units (Lunde *et al.*, 2019). Exposure to occupational kneeling was relatively low in home care, 3.9 min or 0.8% of the workday, likely imposing little risk of health hazards. However, 16% were reporting knee pain. Thus, we cannot rule out the possibility that home care workers being exposed to the highest durations of kneeling, could be at risk of adverse effects.

The considerable variation in work demands depicted in Figs. 3 and 4 is perhaps more noteworthy than the average work demands. The present findings show that 48 (42%) home care workers were having a total activity ranging from 50% to 76% of the workday. Furthermore, that 60 (53%) were sitting from 51% to 92% during working hours, indicating that several workers were exposed to high durations of this physical exposure. Hence, it seems likely that several of these home care workers have an imbalance between physical work demands and rest, as excessive exposure to any physical behavior could induce health problems (Andersen *et al.*, 2016; Holtermann *et al.*, 2019). For instance, too much sedentary time is associated with lifestyle diseases (Patterson *et al.*, 2018), excessive standing could increase the risk of musculoskeletal disorders (Coenen et al., 2018a, b), and high levels of OPA substantially increase the risk of cardiovascular disease and disability retirement due to musculoskeletal pain in the general work-force (Fimland *et al.*, 2018; Holtermann *et al.*, 2021). Considering demanding postures, we found that 75 (66%), 9 (8%), and 5 (4%) home care workers on average were elevating their upper extremities ≥30°, ≥60°, and ≥90° for ≥124 min, ≥37 min, and ≥8 min, respectively. These levels of arm elevation have been associated with a two-fold risk for long-term sickness absence (Gupta *et al.*, 2022). In addition, we found that 5(4%) home care workers were conducting ≥30° forward trunk inclination above 100 min during working hours which has been found to be associated with increased low back pain intensity (Lunde *et al.*, 2019). Although we found that home care workers on average were within the recommended threshold by the ILO (33% HRR) (Bonjer, 1971; Jørgensen, 1985; Wu and Wang, 2002; Brighenti-Zogg *et al.*, 2016), 29% of the workers were above this threshold, and could be at risk of impaired health. Moreover, 25% were spending approximately 50–93% of the workday above 33% HRR, indicating that these workers are exposed to high durations of cardiovascular workload during working hours. Even though the validity of ILO’s threshold for cardiovascular strain is questionable, it is currently the only available attempt at an objectively derived threshold using device-based measurements.

In line with the ‘Goldilocks Work principle’ (Holtermann *et al.*, 2019), there may be several preventive measures in addition to standard ergonomic approaches that could be considered to achieve a healthier balance between physical work demands and rest. In home care, the geographical location of patients varies. Whereas some are in proximity and are easily accessible on foot, others are only accessible through transportation by car. Hence, it could be possible to create a system that provides a balance between active and passive transportation among home care workers. Another possibility could be to modify work lists. For example, all patients in home care have a functioning score. It could be possible to create balanced work lists with ‘heavy’, ‘medium’, and ‘light’ patients. Regardless of the approach chosen, it is important that a participatory approach including both management and home care workers is used, so that work productivity and quality are not compromised.

Some limitations should be acknowledged in this study: First, 58% of eligible home care workers completed the study. Thus, we cannot exclude the possibility of selection bias, as it is plausible that workers not willing to participate had poorer health and different demographics. Second, the participants were only recruited from Trondheim municipality, meaning that our results are not necessarily generalizable to the rest of Norway. However, the organization of home care services is rather homogenous. Third, currently available technical measurement systems feasible for long-term workplace investigations are not capable of detecting demands related to muscle torque and trunk rotation. Fourth, accelerometers were only mounted on one side of the body which could lead to cases of demanding postures not being classified but adding several accelerometers would be quite inconvenient for participants.

## Conclusion

This observational study presents detailed information on device-based measured physical work demands of home care workers in Norway. Home care workers spent on average half the workday sitting and the remaining time in various occupational physical activities and demanding postures. Currently, few device-based exposure limits have been proposed for acceptable amounts of occupational physical exposures, but the level of arm-elevation, forward trunk inclination, and the considerable variation of physical workloads among home care workers, indicate that preventive measures are required. These findings extend our understanding of occupational ergonomic risk factors and can contribute to improved and more tailored occupational health and safety measures in home care, ultimately reducing long-term sickness absence and early retirement, allowing home care workers to stay productive longer through the design of a safer and better workplace. The results can also inform targeted interventions aiming to reduce awkward work positions or evening out workloads between home care workers.

## Data Availability

Collected data and research protocol are available from the corresponding author on reasonable request.
