# Correction to: Assessment of Physical Work Demands of Home Care Workers in Norway: An Observational Study Using Wearable Sensor Technology

**DOI:** 10.1093/annweh/wxac073

**Published:** 2022-11-10

This is a correction to: Svein O Tjøsvoll, Øystein Wiggen, Victor Gonzalez, Trine M Seeberg, Skender Elez Redzovic, Ingeborg Frostad Liaset, Andreas Holtermann, Marius Steiro Fimland, Assessment of Physical Work Demands of Home Care Workers in Norway: An Observational Study Using Wearable Sensor Technology, Annals of Work Exposures and Health, 2022, wxac052, https://doi.org/10.1093/annweh/wxac052

In the originally published version of this manuscript, there was an error in the Materials and methods section. It was stated that “The data were normalized to an average working day of 7.5h”. This was incorrect and the sentence has been removed. Results were normalized by calculating each worker’s weighted mean, as stated in Statistics.

